# Robotic excision of vaginal pouch in ovotesticular disorder of sexual development

**DOI:** 10.1590/S1677-5538.IBJU.2022.0038

**Published:** 2022-05-10

**Authors:** Prashant Singh, Sanjay Kumar, Sridhar Panaiyadiyan, Prem Nath Dogra

**Affiliations:** 1 All India Institute of Medical Sciences Department of Urology New Delhi India Department of Urology, All India Institute of Medical Sciences, New Delhi, India; 2 Sir Ganga Ram Hospital Department of Urology New Delhi India Department of Urology, Sir Ganga Ram Hospital, New Delhi, India

## Abstract

**Purpose:**

Ovotesticular disorder of sexual development (DSD) is the rarest of DSDs with an incidence of 1:20000 (1). Management of vaginal pouches in such cases is warranted for symptomatic presentations and laparoscopy is considered the gold standard treatment (2). We report a rare case of robotic excision of a large symptomatic vaginal pouch in a 19-year-old boy with ovotesticular DSD.

**Material and Methods:**

A 19-year-old boy with ovotesticular DSD post hypospadias repair in early childhood presented with complaints of recurrent UTIs, ballooning of urethra during micturition and post-void dribbling. Ultrasound, voiding cystourethrogram (VCUG) and magnetic resonance imaging (MRI) were suggestive of a vaginal pouch. The patient underwent endo-evaluation followed by robot-assisted excision of the vaginal pouch. Endo-evaluation showed two orifices in the posterior urethra. The posterior orifice was leading into a blind-ending rudimentary uterus and the true urethra was lying anteriorly. The DaVinci Xi Robotic Surgical System was used and the entire pouch was dissected free of the surrounding tissues using monopolar scissors. The pouch was transected just a few millimetres from its junction with the urethra. The urethra was then closed with V-loc 4-0 suture. The patient was discharged on postoperative day 2 and the catheter was removed on day 21.

**Results:**

Follow-up VCUG at 6 weeks did not show any residual pouch. There was no complaint of post-void dribbling or UTI at 30 months of follow-up.

**Conclusion:**

Robot-assisted laparoscopy should be considered as an alternative to laparoscopy for the primary treatment of a large symptomatic vaginal pouch.
